# Trigger-Based Referral and Needs Assessment in the Intensive Care Unit: A Narrative Review and Conceptual Synthesis

**DOI:** 10.3390/healthcare14193199

**Published:** 2026-09-28

**Authors:** Majid Golestani, Georg Bollig

**Affiliations:** 1 Department of Anaesthesiology and Critical Care, Helios Klinikum Schleswig, St. Jürgener Straße 1–3, 24837 Schleswig, Germany; 2 Department of Palliative Medicine, Faculty of Medicine and University Hospital, University of Cologne, 50924 Cologne, Germany; georg.bollig@helios-gesundheit.de

**Keywords:** intensive care unit, palliative care, trigger-based referral, needs assessment, critical illness, family-centered care, shared decision-making, primary palliative care, specialist palliative care, end-of-life care

## Abstract

**Highlights:**

**What are the main findings?**

- Trigger-based referral criteria provide a practical way to identify clinical vulnerability, but their relationship with directly measured multidimensional palliative care need remains uncertain.
- One multicenter study found limited discrimination of serious unmet need by conventional trigger status; replication is required across populations, instruments, and healthcare systems.

**What are the implications of the main findings?**

- Trigger criteria should remain available as one route to further assessment rather than being treated as validated measures of need or automatic mandates for specialist referral.
- Prospective studies should compare trigger-only, needs-assessment, integrated, and usual-care strategies before any combined approach is adopted.

**Abstract:**

**Background/Objectives**: Trigger-based referral systems are widely used to identify intensive care unit (ICU) patients who may benefit from palliative care, but the extent to which trigger status corresponds to multidimensional patient and family need remains uncertain. This narrative review examines trigger-based referral, needs assessment, and integrated approaches to ICU palliative care. **Methods**: PubMed/MEDLINE, Scopus, Web of Science, Google Scholar, and reference lists were searched, and a purposive thematic synthesis was conducted. The original identification-stage record counts were unavailable; selection was reconstructed from an archived full-text library, and screening was primarily performed by one author. **Results**: Trigger-based systems improved recognition of clinically vulnerable patients and several care processes. Direct evidence comparing trigger status with measured need is limited, however, and derives principally from one multicenter study using a family-member-reported NEST score. The literature supports distinguishing clinical risk from measured need but does not establish the superiority of trigger-based, needs-based, or integrated models. **Conclusions**: Current evidence supports comparative testing rather than adoption of any integrated configuration. Candidate components, timing, thresholds, feasibility, and applicability across healthcare systems require prospective evaluation.

## 1. Introduction

The integration of palliative care into intensive care medicine has undergone substantial transformation over the past two decades. Traditionally, palliative care in the intensive care unit (ICU) was closely associated with end-of-life care, withdrawal of life-sustaining treatment, and decision-making in patients with irreversible illness [1,2,3]. Consequently, early models of ICU palliative care were largely reactive, with specialist involvement often occurring only after curative or life-prolonging treatment options had been exhausted [1,2,4]. Over time, however, this approach has been increasingly challenged by the recognition that the needs of critically ill patients and their families extend far beyond the final stages of life and frequently arise throughout the trajectory of critical illness [5,6].

Critically ill patients commonly experience a wide range of distressing symptoms, including pain, dyspnea, delirium, agitation, anxiety, thirst, sleep disturbance, and communication difficulties, even when meaningful recovery remains possible [6,7]. At the same time, family members are often confronted with uncertainty, emotional distress, surrogate decision-making responsibilities, and the risk of long-term psychological sequelae, particularly in the context of prolonged mechanical ventilation or chronic critical illness [8,9,10]. Many of these challenges emerge early during ICU admission and may persist long after discharge, contributing substantially to the overall burden of critical illness for both patients and families [6,10].

Advances in modern intensive care have further increased the relevance of palliative care within critical care practice. Contemporary ICUs can sustain organ function in patients with profound physiological instability, yet prognostic uncertainty frequently remains substantial despite increasingly sophisticated diagnostic and therapeutic technologies [1,5]. Decisions regarding the initiation, continuation, escalation, or limitation of life-sustaining therapies are therefore often made in the setting of evolving illness trajectories and incomplete knowledge of patient preferences and values [11,12]. Although advance care planning and advance directives may provide important guidance in such situations, these documents are frequently unavailable, outdated, or insufficiently specific to inform complex treatment decisions in the ICU [12,13]. As a result, palliative care is increasingly viewed not as an alternative to intensive care but as an essential component of high-quality critical care that supports symptom management, communication, shared decision-making, and goal-concordant treatment throughout the course of serious illness [5,13].

Initial efforts to improve palliative care integration relied largely on specialist consultation initiated at the discretion of individual clinicians [2,4]. Concerns regarding delayed referrals, inconsistent consultation practices, and variability in access to specialist expertise subsequently led to the development of trigger-based referral systems. These systems used predefined clinical criteria—such as advanced malignancy, prolonged mechanical ventilation, recurrent ICU admission, multisystem organ failure, or prolonged ICU length of stay—to identify patients who might benefit from specialist palliative care involvement [14,15]. In many settings, trigger-based approaches improved recognition of potentially eligible patients, facilitated goals-of-care discussions, and increased consultation rates [14,15]. Nevertheless, accumulating evidence has suggested that prognostic risk, illness severity, and healthcare utilization may not reliably reflect the actual supportive care needs of patients and families [16,17]. Individuals experiencing significant symptom burden, communication difficulties, psychological distress, decisional conflict, or uncertainty may remain unrecognized despite not meeting established trigger criteria [7,16,17].

These observations have prompted growing interest in more individualized and needs-based models of palliative care integration. At the same time, there has been increasing recognition that many palliative care needs can be addressed directly by ICU clinicians and do not necessarily require specialist consultation [13,18]. This shift has contributed to the emergence of primary palliative care as a core competency within critical care practice. Contemporary models increasingly combine structured screening with ongoing reassessment of symptoms, communication needs, patient values, prognostic uncertainty, and evolving treatment goals throughout the ICU stay [18,19,20,21]. Within such frameworks, specialist palliative care consultation is reserved for patients and families with particularly complex needs, while routine palliative care activities are integrated into everyday ICU practice [13,18].

Despite the widespread adoption of trigger-based referral systems, the empirical relationship between trigger status and directly measured palliative care need remains uncertain. Triggers may identify clinical vulnerability efficiently, yet this does not necessarily establish which patients or families have the greatest symptom, communication, psychological, decisional, social, or spiritual needs. Equally, direct needs assessment has its own measurement and implementation limitations.

Three issues remain unresolved. First, direct comparisons between trigger status and measured palliative care need are scarce and are dominated by one multicenter study. Second, trigger-based screening, primary palliative care, and needs assessment have often been discussed separately, leaving uncertainty about how they might relate operationally. Third, the feasibility and transferability of integrated approaches remain largely untested, particularly outside well-resourced North American and Western European systems. The value of this review lies in clarifying these uncertainties and identifying testable questions rather than asserting a preferred model of care.

In this narrative review, we synthesize and critically evaluate the literature comparing trigger-based and needs-based approaches to palliative care identification in adult ICUs. We distinguish empirically supported findings from author-generated propositions and outline candidate components for future comparative research in the Supplementary Materials.

## 2. Materials and Methods

### 2.1. Study Design

This article was designed as a narrative review examining the conceptual and clinical evolution of palliative care integration within the intensive care unit (ICU), with particular attention to trigger-based referral systems, needs-based approaches, and emerging hybrid models of care. Because the objective was to provide a critical and interpretive synthesis of a conceptually heterogeneous literature rather than a quantitative summary of intervention effects, a narrative review methodology was considered the most appropriate approach. The review was conducted according to the methodological recommendations for narrative literature reviews described by Green et al. [22] and reported in accordance with the key principles of the Scale for the Assessment of Narrative Review Articles (SANRA) [23], including justification of the review type, transparent description of the literature search, balanced presentation of the evidence, and explicit consideration of limitations. Because this review was designed as a narrative synthesis rather than a systematic review, protocol registration, duplicate independent screening, formal risk-of-bias assessment, and quantitative meta-analysis were not performed.

### 2.2. Search Strategy

A structured literature search was performed in PubMed/MEDLINE, Scopus, and Web of Science, covering the period from 1 January 2000 to 30 April 2026. Google Scholar was used as a supplementary source to identify additional publications, including conceptual papers, consensus statements, and grey literature not indexed in the primary databases. The original database search was last performed in April 2026.

The search strategy combined three conceptual blocks—(i) the critical care setting, (ii) palliative and supportive care, and (iii) models of integration and assessment—linked with the Boolean operator AND, while terms within each block were combined with OR. The core search string, adapted to the syntax and controlled vocabulary of each database (including MeSH terms in PubMed/MEDLINE where applicable), was as follows:

(“intensive care unit*” OR “critical care” OR “ICU” OR “critically ill”) AND (“palliative care” OR “end-of-life care” OR “supportive care” OR “comfort care”) AND (trigger* OR “referral criteria” OR “consultation criteria” OR “screening criteria” OR “needs assessment” OR “needs-based” OR “primary palliative care” OR “specialist palliative care” OR “family-centered care” OR “family-centred care” OR “goals of care” OR “shared decision-making” OR communication OR “chronic critical illness”).

For Google Scholar, which does not support complex Boolean nesting, simplified combinations of the same key terms were used (e.g., “intensive care” “palliative care” trigger; “ICU” “palliative care” “needs assessment”). In addition, the reference lists of key reviews, clinical practice guidelines, consensus statements, landmark trials, and major conceptual papers were screened manually to identify further relevant publications (backward citation searching).

As a focused sensitivity check, PubMed/MEDLINE was searched again on 18 September 2026 using the following exact query:

((“intensive care unit”[Title/Abstract] OR “critical care”[Title/Abstract] OR ICU[Title/Abstract]) AND “palliative care”[Title/Abstract] AND (trigger*[Title/Abstract] OR “screening criteria”[Title/Abstract]) AND (need*[Title/Abstract] OR “needs assessment”[Title/Abstract]) AND (“1 January 2000”[Date-Publication]: “30 April 2026”[Date-Publication])).

The search retrieved 47 records. Title-and-abstract assessment identified no additional study that directly compared conventional trigger status with contemporaneously measured multidimensional patient or family need in a general adult ICU population beyond the Cox et al. cohort [17]. This retrospective, single-database check was intended to test the sensitivity of the central empirical claim; it does not establish comprehensiveness and was not used to recast the review as systematic.

Three recent adjacent publications were examined separately because they could appear relevant to this narrow claim. Hou et al. synthesized trials of automated electronic health record algorithms and included NEST as an intervention outcome, but did not compare individual trigger classification with contemporaneous multidimensional need [24]. Tenge et al. developed the PC-ICU score against receipt of specialist palliative care consultation, rather than against a multidimensional need measure [25]. Hochberg et al. evaluated implementation of early screening against consultation uptake and utilization outcomes, without measuring multidimensional need [26]. None therefore met the prespecified narrow criterion for a direct trigger-versus-need comparison.

### 2.3. Eligibility Criteria and Study Selection

Publications were considered for inclusion if they (i) addressed palliative care integration in adult ICU or critical care populations directly, or provided a clearly transferable conceptual, communication, or implementation contribution to the ICU-focused questions of this review; (ii) focused on topics directly relevant to the review objectives, including consultation models, trigger-based screening strategies, needs-based and hybrid approaches, communication interventions, symptom burden, family-centered outcomes, chronic critical illness, primary and specialist palliative care, or implementation challenges; and (iii) were published in English between 1 January 2000 and 30 April 2026. Eligible source types comprised original research articles (randomized controlled trials, observational studies, qualitative investigations, and implementation studies), systematic reviews, consensus statements, clinical practice guidelines, influential conceptual publications, and authoritative organizational sources used for definitions or contextual framing. The full texts of six included publications [7,27,28,29,30,31] were assessed during the original synthesis, but their corresponding PDF copies are no longer present in the surviving archive. For the reconstructed audit, their bibliographic details, designs, and contributions were re-verified against indexed records and published abstracts, and only information independently verifiable from those records was retained in the evidence matrix. The World Health Organization source [32] was verified from its official web page and had never been archived as a PDF. These archive exceptions are identified in Supplementary Tables S1 and S2.

Publications were excluded as outside the prespecified synthesis scope if they (i) focused exclusively on non-ICU settings without a direct and clearly transferable contribution to the ICU integration questions; (ii) addressed exclusively pediatric or neonatal populations; (iii) were protocols without results; (iv) reported cohorts defined by a single condition, such as COVID-19-specific cohorts, that did not address the comparative integration-model question; (v) were descriptive practice surveys or regional status reports without comparative or conceptual content bearing on models of integration; or (vi) focused on a narrow clinical domain, such as a single symptom or a single procedural or ethical process, that was peripheral to the identification and integration question. For studies involving mixed populations, only findings directly applicable to adult critical care were considered.

Study selection proceeded in two stages. Titles and abstracts were screened against the eligibility criteria, followed by full-text assessment of potentially relevant publications. Screening and selection were performed by the first author (M.G.); uncertain cases were discussed with the second author (G.B.) until consensus was reached. Identification-stage database counts were not documented prospectively, so the completeness of the original search cannot be determined.

Of the 78 files reported in the previous revision, six are no longer present in the surviving archive (file identifiers 8 and 36–40). The remaining numbered files total 72, and an additional copy stored as Article (60) [1] yields 73 surviving PDF files. The six absent files account for six of the seven included sources without a corresponding archived PDF; the seventh is the official World Health Organization web source, which was never archived as a PDF. Bibliographic and file-level comparison identified 9 duplicate copies among the 73 surviving files, leaving 64 unique archived publications. Two unrelated archiving errors were then removed, leaving 62 unique archived publications for relevance assessment. Eighteen were excluded because they met one or more prespecified scope criteria. Of 44 eligible archived publications, 17 were not selected for the purposive synthesis only when their substantive contribution was represented by a broader retained source; had been superseded or represented by a later review, larger study, or later analysis; or added no distinct incremental information to the review questions. These conditions are coded in Supplementary Table S2 as A (contribution represented by a broader retained source), B (superseded or represented by a later review, larger study, or later analysis), and C (commentary, letter, or conceptual item with no distinct incremental information). The codes are decision categories, not quality grades. Twenty-seven archived publications were retained. Seven additional included sources had no corresponding PDF in the surviving archive, producing a final synthesis set of 34 publications. Supplementary Table S2 documents every duplicate, archiving error, scope exclusion, non-selection decision, and archive exception. No publication was set aside because of the direction of its findings, and no non-selected publication reported findings that contradicted a conclusion of this review (Figure 1).

The final integration-model synthesis comprised 34 publications: 27 retained from the reconstructed archive and 7 additional cited sources not represented by a surviving archived PDF. Two methodological references [22,23] informed review design but were not part of the evidence synthesis. Instrument-development and measurement-validation sources cited in Section 3.5 were used to characterize measurement properties and were not counted as integration-model studies. Such contextual measurement sources were not subject to the 2000–2026 date restriction applied to integration-model studies; this accounts for the 1991 instrument source [38]. Publications [24,25,26] are cited only to document the focused confirmatory sensitivity check and were not added to the 34-publication synthesis. Because the original identification stage was not documented and the surviving archive was demonstrably incomplete, this review cannot be interpreted as an exhaustive or fully reproducible systematic account of the literature.

### 2.4. Data Extraction and Organization

For each included publication, the following information was extracted: bibliographic details (authors, year, country/setting); study design and publication type; population and clinical context; the model of palliative care integration examined (consultative, trigger-based, needs-based, primary, or hybrid); the specific trigger criteria or needs-assessment instruments described; reported patient-, family-, and process-related outcomes; principal findings; and reported implementation facilitators and barriers. Data extraction was performed by the first author (M.G.) and organized in a structured summary matrix (spreadsheet format), in which publications were grouped according to the conceptual domain(s) they addressed. The extracted material and its thematic assignment were reviewed by the second author (G.B.), and discrepancies were resolved by discussion.

### 2.5. Data Synthesis and Analysis

The evidence was synthesized using a thematic, interpretive approach appropriate to narrative review methodology. Publications were read in full and grouped iteratively around four principal themes: the rationale for palliative care integration; the development and achievements of trigger-based referral; the relationship between trigger status and measured need; and needs assessment, primary palliative care, and integrated approaches. Two cross-cutting themes addressed implementation and applicability across healthcare systems. Theme development was led by the first author and critically reviewed by the second author. No quantitative pooling or formal risk-of-bias assessment was performed. Key characteristics of the 34 included publications are summarized in Supplementary Table S1.

Because the synthesized literature comprised heterogeneous publication types, the nature and strength of the underlying evidence were explicitly considered during synthesis and interpretation. Findings from randomized trials and prospective observational studies were treated as empirical evidence of effects or associations; qualitative and implementation studies were used to characterize acceptability, feasibility, and contextual factors; and clinical practice guidelines, consensus statements, and conceptual publications were treated as expert recommendations rather than as independent empirical verification of effectiveness. Conclusions regarding clinical effectiveness were anchored to empirical studies, and the Discussion distinguishes empirically supported findings from conceptual inference.

## 3. Results

The synthesis is organized around four principal themes and two cross-cutting implementation themes.

### 3.1. Why Palliative Care Belongs in the Intensive Care Unit

The rationale for integrating palliative care into routine ICU practice extends far beyond traditional end-of-life care and symptom management. Modern ICUs increasingly care for patients with severe symptom burden, prolonged physiological instability, uncertain illness trajectories, and complex decisional needs that frequently coexist with aggressive life-sustaining therapies [1,5,7]. Consequently, palliative care has evolved from a service primarily associated with dying patients to a broader supportive framework that also encompasses patients with chronic life-limiting diseases, aiming to improve symptom control, communication, decision-making, and alignment of treatment with patient-centered goals throughout the course of critical illness [5,10,40].

Physical suffering remains common among critically ill patients and is often difficult to recognize. Pain, dyspnea, thirst, agitation, delirium, anxiety, sleep disturbance, and communication difficulties frequently occur in both mechanically ventilated and non-ventilated patients [7,40]. Symptom assessment is further complicated by sedation, neurologic dysfunction, and impaired communication, increasing the risk of unrecognized distress. Studies have consistently demonstrated that significant physical and psychological symptoms may persist despite ongoing disease-directed treatment, highlighting that palliative care and intensive care are complementary rather than competing approaches [5,19,41].

Palliative care needs, however, are not confined to the patient. Palliative care is inherently family-centered care, encompassing both the patient and the family. Family members are frequently required to participate in complex surrogate decision-making under conditions of uncertainty, emotional distress, and evolving prognostic information [6,8,10]. Anxiety, depression, post-traumatic stress symptoms, decisional conflict, and prolonged bereavement-related distress are common among relatives of critically ill patients [8,10]. Multiple communication-focused interventions have demonstrated that structured family support and proactive clinician–family communication can improve important patient- and family-centered outcomes, reinforcing the central role of communication as a therapeutic intervention within critical care practice [8,27,40].

Advances in organ support technologies have further expanded the relevance of palliative care in critical care medicine. While short-term survival has improved, increasing numbers of patients experience chronic critical illness, prolonged mechanical ventilation, frailty, cognitive impairment, functional disability, and persistent symptom burden after ICU discharge [10,12,13]. These realities have challenged the traditional reliance on mortality as the dominant outcome measure and have shifted attention toward quality of life, functional recovery, treatment burden, and goal-concordant care [19,28].

Despite broad agreement regarding the importance of palliative care in the ICU, an important practical question remains: how can clinicians reliably identify patients and families who would benefit most from additional palliative care support? The search for an operational answer to this question provided the foundation for the development of trigger-based referral systems, which subsequently became one of the dominant models of palliative care integration in critical care practice.

### 3.2. The Emergence of Trigger-Based Referral Systems

As awareness of unmet palliative care needs in the ICU increased, clinicians and healthcare systems faced a practical challenge: identifying which patients and families would benefit from specialist palliative care consultation. Reliance on individual clinician judgment alone often resulted in delayed, inconsistent, and highly variable referral practices, with consultations frequently occurring late in the course of illness or only after decisions regarding treatment limitation had already been made [2,11].

To address this problem, several groups proposed the use of predefined referral criteria, commonly referred to as trigger-based screening systems. The underlying rationale was straightforward: if patients at increased risk of mortality, prolonged critical illness, or complex care needs could be identified early through objective clinical criteria, specialist palliative care consultation could be initiated in a more timely and systematic manner [14,15].

Early trigger models typically relied on diagnosis-based, prognosis-based, or utilization-based criteria. Common triggers included advanced malignancy, prolonged mechanical ventilation, recurrent ICU admission, multisystem organ failure, prolonged ICU length of stay, severe neurological injury, advanced age, and post-cardiac arrest status [14,15,29]. These criteria were intentionally designed to be simple, reproducible, and easily incorporated into routine clinical workflows.

The Improving Palliative Care in the ICU (IPAL-ICU) initiative played a particularly influential role in the development of trigger-based referral strategies. Nelson and colleagues proposed structured screening criteria intended to identify critically ill patients who were likely to benefit from specialist palliative care consultation and provided practical guidance regarding implementation within diverse ICU settings [4,14]. Their work helped shift palliative care integration from a purely reactive process toward a more proactive and operationally feasible model.

Subsequent observational studies suggested that trigger-based approaches could identify a substantial proportion of ICU patients with potentially unmet palliative care needs. Using a large multicenter dataset, Hua and colleagues estimated that approximately 14–20% of ICU patients would meet commonly used trigger criteria for specialist palliative care consultation, depending on the specific trigger set applied [15]. These findings suggested that trigger systems could provide a scalable method for identifying high-risk patients in busy critical care environments.

Beyond improving consultation rates, trigger-based programs were also associated with several clinically relevant process outcomes. Studies reported improvements in goals-of-care discussions, advance care planning, code-status documentation, hospice referral, and, in some settings, reductions in ICU length of stay or treatment intensity among selected patient populations [18,27,42,43]. These observations reinforced the perception that trigger-based referral systems could serve as an effective bridge between critical care and specialist palliative care services.

Importantly, trigger-based models emerged during a period when specialist palliative care resources were limited and awareness of palliative care within critical care medicine remained relatively low [1,2]. Under these circumstances, standardized referral criteria provided a pragmatic mechanism for promoting earlier consultation and reducing dependence on individual clinician preferences. Consequently, trigger-based screening became one of the most widely adopted and extensively studied approaches to ICU palliative care integration.

Despite these important contributions, trigger-based systems were primarily designed to identify patients at increased clinical risk rather than to directly measure the multidimensional needs of patients and families. As the field matured, questions increasingly emerged regarding whether trigger status truly reflected palliative care need or merely identified populations with a higher probability of serious illness. These concerns ultimately laid the foundation for a critical reappraisal of trigger-based referral models and the subsequent development of needs-based approaches. Key milestones in this evolution are summarized in Table 1 and illustrated in Figure 2.

### 3.3. Strengths and Achievements of Trigger-Based Models

Trigger-based referral systems addressed important operational barriers to palliative care integration. Before structured screening, specialist consultation often depended on individual clinician recognition and occurred variably or late in the illness trajectory [2,11]. Predefined criteria offered a reproducible way to identify patients with advanced illness, prolonged organ support, recurrent admission, or high predicted mortality [14,15,16].

Their principal strengths are simplicity, consistency, and potential automation within electronic health records. Trigger programs have increased consultation rates and have been associated with earlier goals-of-care discussions, improved documentation, hospice transitions, and selected utilization outcomes, although patient- and family-reported benefits remain inconsistent [18,29,42,43].

Triggers may be particularly useful where specialist teams need transparent criteria to prioritize a limited service. Their value therefore extends beyond prediction of need: they can standardize workflow, reduce reliance on individual referral habits, and create opportunities for earlier assessment [14,15].

These operational benefits should not be conflated with validated measurement of multidimensional need. Most criteria were developed around diagnosis, prognosis, organ dysfunction, or resource use rather than direct assessment of symptoms, communication, family burden, or decisional conflict [14,15,16].

### 3.4. Trigger Status and Measured Palliative Care Need

Trigger status and multidimensional palliative care need are conceptually related but non-equivalent constructs. Clinical risk may increase the probability of supportive care need, yet patients with similar illness severity can differ in symptom burden, communication, decisional conflict, family distress, and cultural or spiritual concerns [14,15,16].

Evidence directly testing this distinction is limited. The principal study is the multicenter prospective cohort by Cox and colleagues, in which conventional trigger status showed limited discrimination for serious unmet need reported by family members using the Needs Near the End-of-Life Care Screening Tool (NEST; termed the Needs at the End-of-Life Screening Tool in the Cox report), with a c-statistic close to 0.50 [17,33]. This finding questions the use of trigger status as a stand-alone proxy for need but does not invalidate trigger-based referral as an operational strategy.

Generalizability remains uncertain because the direct comparison derives largely from one United States study using one needs-assessment instrument and one set of trigger definitions [17]. Different populations, instruments, trigger sets, and healthcare systems may produce different results. Qualitative work also indicates that clinicians value the consistency of triggers while expressing concern about over-referral, local fit, and specialist capacity [44].

Systematic review evidence further shows substantial heterogeneity in trigger definitions, referral processes, and outcome selection [29]. These findings support caution about treating any single trigger set as universally valid, but they do not demonstrate that needs-based referral is superior.

### 3.5. Needs Assessment and Primary Palliative Care

#### 3.5.1. Rationale and Conceptual Appeal

Needs assessment offers a complementary approach by examining symptoms, communication, psychological distress, family burden, decisional conflict, and social or spiritual concerns directly rather than inferring them from clinical risk. The World Health Organization definition of palliative care supports this multidimensional orientation [32]. Its conceptual appeal is that it addresses the construct of interest rather than a correlate of it: whereas triggers ask who is likely to need support, needs assessment asks who reports needing it.

This appeal, however, should not be mistaken for demonstrated measurement adequacy in critical care. Direct assessment substitutes one set of measurement assumptions for another, and in the ICU those assumptions are demanding (Table 2).

#### 3.5.2. Measurement Properties and Limitations in the ICU

Five limitations recur across instruments relevant to multidimensional needs assessment in critical care (Table 3).

Provenance and validation context. Most multidimensional needs instruments were developed and validated in ambulatory oncology, hospice, or general palliative care populations rather than in critical care [33,34]. Psychometric properties established in communicative, cognitively intact populations cannot be assumed to transfer to sedated, mechanically ventilated patients with fluctuating consciousness. ICU adaptation has generally been pragmatic rather than supported by formal ICU-specific validation [17,21].

Respondent capacity. Sedation, delirium, neurological injury, and endotracheal intubation make reliable self-report impossible for a substantial proportion of ICU patients [7,40]. This is a structural constraint: patients most likely to meet conventional clinical triggers are often those least able to complete a multidimensional self-report instrument.

Proxy reporting. Where patients cannot respond, family members or clinicians serve as proxies. In a prospective ICU study, patient–family agreement reached the prespecified acceptable threshold for symptom intensity in only 6 of 10 symptoms; agreement was poorer for clinician proxies, who frequently overestimated or underestimated patient-reported intensity and distress [35]. Proxy reports may also conflate the patient’s experience with the proxy’s own distress, while family absence creates non-random missingness related to social support, distance, employment, and language.

Absence of validated action thresholds. Needs instruments yield scores or domain profiles; they do not establish what magnitude or pattern of need should prompt specialist consultation rather than management by the ICU team. Thresholds used in practice have generally been selected pragmatically rather than calibrated against patient outcomes [17,20]. A needs score used as a referral rule therefore introduces an unvalidated decision threshold, just as a trigger set does.

Temporal properties. Need in critical illness is dynamic, whereas most multidimensional instruments were designed for cross-sectional or slower-changing settings. Responsiveness to clinically meaningful change, minimal important difference, optimal reassessment interval, and stability across an ICU trajectory remain poorly characterized [20,21,34].

#### 3.5.3. Implications for Interpreting Trigger-Need Comparisons

Instrument-derived need is not a gold standard against which trigger performance can be definitively scored. It is an imperfect operationalization of a latent multidimensional construct, affected by respondent capacity, proxy substitution, domain coverage, and threshold selection. A weak association between trigger status and instrument-measured need is therefore compatible with several interpretations: triggers may identify a different construct, the instrument may incompletely capture need in this population, or both. Discordance between two imperfect measures identifies a problem but does not by itself identify which measure is at fault [17,35].

This interpretation constrains the inference from Section 3.4. The Cox study supports the conclusion that trigger status should not be treated as a validated proxy for need; it does not establish direct needs assessment as a superior basis for allocating specialist palliative care.

#### 3.5.4. Primary Palliative Care and Generalist-Plus-Specialist Allocation

Primary palliative care locates basic symptom management, communication, values clarification, family support, and shared decision-making within routine ICU practice [14,41,44]. In the generalist-plus-specialist model, specialist involvement is reserved for complex or refractory symptoms, persistent conflict, or needs that exceed local expertise [41].

This allocation may improve reach where specialist resources are limited, but it assumes that ICU clinicians have appropriate time, training, and institutional support. Those assumptions are not established consistently across settings [18,21]. It also presupposes a reliable mechanism for recognizing when local expertise has been exceeded, a judgment currently supported neither by validated instrument thresholds nor by validated trigger criteria.

Needs assessment is more difficult to automate and standardize than most clinical triggers. Its feasibility, respondent burden, optimal frequency, and effect on outcomes remain uncertain [17,20,21]. It should therefore be considered a complementary measurement strategy with unresolved validity requirements rather than an inherently superior replacement for trigger screening.

### 3.6. Integrated Approaches as a Research Proposition

The literature provides a rationale for testing combinations of trigger-informed screening, direct assessment of need, ICU-delivered primary palliative care, and selective specialist consultation. It does not establish that such combinations improve patient, family, process, or resource outcomes compared with trigger-only, needs-only, or usual-care models [18,19,20,21].

For research purposes, trigger positivity, clinician concern, patient or family request, and clinically meaningful transition can be treated as candidate entry points for assessment. The timing of assessment, escalation thresholds, reassessment schedule, division of responsibilities, and required workforce are deliberately left as variables for prospective calibration rather than specified as practice recommendations (Table 4).

## 4. Discussion

This review supports a distinction between clinical vulnerability and multidimensional palliative care need, but it does not establish that either trigger-based or needs-based identification is superior. Trigger pathways standardized referral and improved several care processes [14,15,16,18,29,42,43]. Direct needs assessment addresses domains that clinical triggers were not designed to measure, but its ICU validity, proxy dependence, action thresholds, temporal responsiveness, feasibility, and effect on outcomes remain unresolved (Section 3.5.2) [17,20,21,33,34,35,36,37,38,39].

The Cox multicenter study provides the clearest direct evidence that conventional trigger status may discriminate poorly between higher and lower instrument-measured need [17]. Its influence on this review must remain limited: it represents one healthcare context, one family-member-reported NEST measure, and one set of triggers. Moreover, instrument-derived need is not a definitive reference standard (Section 3.5.3). Replication may confirm, modify, or refute the observed relationship in other populations and systems.

Primary palliative care and generalist-plus-specialist allocation are supported conceptually and by professional recommendations [4,19,30,41]. The specific sequencing proposed in this review, however, is author-generated. It should be judged by prospective comparison with existing practice, not treated as an evidence-based pathway.

Implementation will depend on instrument burden, clinician training, specialist availability, electronic workflow, culture, and organizational capacity [21,45]. In resource-constrained settings, broad multidimensional assessment and ready specialist escalation may be unrealistic. Brief locally adapted assessments, strengthened primary palliative care skills, and pragmatic clinical triggers may be more feasible, but these strategies also require evaluation. The proposed framework therefore cannot be assumed to transfer unchanged across health systems [31]. Hospital-level palliative care implementation has also been associated with lower ICU use during terminal hospitalizations, although this observational finding does not establish the effect of a specific identification model [46].

Accordingly, the appropriate conclusion is equipoise. Current evidence justifies comparative evaluation of trigger-only, needs-assessment, integrated, and usual-care approaches, with attention to benefits, burdens, unintended effects, specialist use, equity, and cost. It does not justify replacing established trigger pathways on the basis of the presently available direct evidence.

### A Framework for Comparative Evaluation

The preceding sections identify uncertainties rather than a preferred model. Supplementary Figure S1 therefore presents a research architecture, not a clinical algorithm. It assembles established elements into a falsifiable configuration: baseline ICU-delivered primary palliative care, several candidate entry points to assessment, selective direct needs assessment, complexity-guided specialist allocation, and structured reassessment [4,14,18,19,41].

Each component corresponds to a specific uncertainty rather than a claim of optimality. Baseline primary palliative care is included because much palliative care activity is delivered by the ICU team irrespective of referral status. Multiple entry points are included because trigger positivity identifies clinical vulnerability rather than measured need. Needs assessment is selective rather than universal because respondent-capacity, proxy, threshold, and temporal limitations make universal administration unsupported. Reassessment is included because need changes during critical illness, while the temporal properties of both triggers and instruments remain poorly characterized. Complexity-guided allocation is included because specialist capacity is constrained in most systems.

Three alternative configurations were considered. A consultative-only model remains an appropriate comparator but is associated with variable and sometimes late involvement [2,11]. A fully integrative model may be preferable where staffing and training permit structured palliative care for all ICU patients, but that capacity cannot be assumed across settings [21,45]. A needs-assessment-first model would become more defensible if instruments with demonstrated ICU validity and empirically derived action thresholds became available.

The architecture deliberately leaves timing, instrument choice, escalation thresholds, division of responsibility, and workforce requirements unspecified because these are empirical parameters. No comparative evidence shows that this or any other integrated configuration outperforms trigger-only, needs-only, or usual care. It is presented in the Supplementary Materials to make assumptions explicit for prospective testing, not to imply validation.

## 5. Limitations

Several limitations of this narrative review should be acknowledged.

First, this was a purposive narrative synthesis rather than a systematic review. It had no predefined protocol, formal risk-of-bias assessment, or prospective record of the database-identification stage. Of the 78 files reported previously, six are no longer present (file identifiers 8 and 36–40); the surviving archive therefore contains 73 files when the additional Article (60) [1] copy is counted. Removal of 9 duplicate copies yielded 64 unique archived publications, and removal of 2 unrelated archiving errors left 62 publications for relevance assessment. Seven included sources have no corresponding PDF in the surviving archive, confirming that the archive is incomplete. The focused confirmatory PubMed search reduces the risk that an additional direct trigger-versus-need comparison was missed, but its retrospective, single-database, single-reviewer nature does not establish overall search comprehensiveness. Author-driven selection and interpretation bias therefore remain possible despite the operational rules and publication-level audit trail.

Second, screening and extraction were led by one reviewer, with the second author resolving uncertain cases and reviewing extracted material rather than independently duplicating all procedures. English-language restriction may have excluded relevant evidence. The full texts of six included publications were assessed during the original synthesis, but their archived PDF copies are no longer available. For the present reconstruction, their bibliographic details and contributions were re-verified against indexed records and published abstracts, and only independently verifiable information was retained. One organizational source was verified from its official web page. Overlap between primary studies and reviews may also have amplified some themes.

Third, the central empirical claim concerning discordance between conventional triggers and measured need depends largely on one multicenter United States cohort using one instrument and one trigger set [17]. This finding may not be reproducible in other populations or healthcare systems and should be treated as preliminary.

Fourth, substantial heterogeneity in referral criteria, interventions, implementation, and outcomes limits comparison. Much of the literature emphasizes process or utilization outcomes, whereas evidence on symptoms, goal concordance, decisional quality, family experience, and long-term outcomes is limited.

Fifth, most evidence originates from North American and Western European systems. Specialist availability, ICU staffing, cultural expectations, and infrastructure differ substantially elsewhere, making the applicability of all reviewed models—including the proposed framework—uncertain.

Finally, the framework is an author-generated synthesis, not a validated model. Its entry points, timing, thresholds, responsibilities, resource effects, and potential unintended consequences must be determined empirically before clinical use.

## 6. Future Directions

Future studies should prospectively compare trigger-only, direct needs-assessment, integrated, and usual-care strategies. Priority outcomes include symptom burden, goal concordance, decisional quality, family experience, clinician workload, specialist utilization, equity, cost-effectiveness, and unintended effects.

Research should test alternative trigger sets and needs instruments across healthcare systems, including resource-constrained settings. Assessment timing, escalation thresholds, reassessment rules, workforce requirements, cultural adaptation, and electronic integration should be treated as variables to be calibrated rather than predetermined features of a model.

## 7. Conclusions

Trigger-based pathways provide a practical and reproducible method for identifying clinical vulnerability, but direct evidence linking trigger status to multidimensional palliative care need is limited. One multicenter study raises an important question about this relationship; it does not establish the general superiority of needs-based or integrated approaches.

The candidate components described in “A Framework for Comparative Evaluation” and Supplementary Figure S1 are a research proposition, not a validated model. Comparative evaluation across diverse healthcare systems is required before any configuration can be translated into practice recommendations.

## Figures and Tables

**Figure 1:**
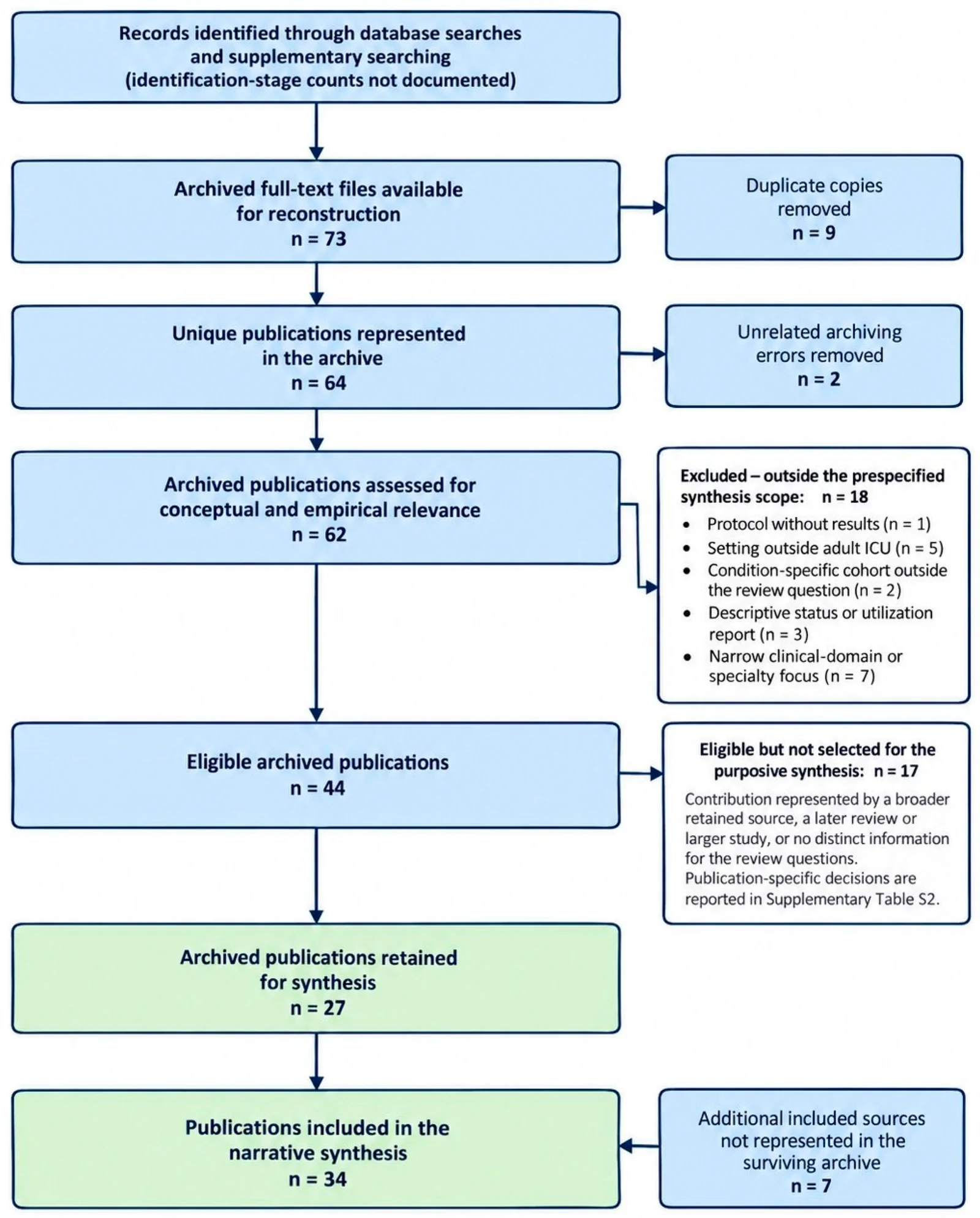
Reconstructed study-selection flow diagram. Identification-stage database counts were not documented during the original search. Of the 78 files reported previously, six are no longer present (file identifiers 8 and 36–40); 72 remaining numbered files plus an additional copy stored as Article (60) [1] yielded 73 surviving PDF files. Removal of 9 duplicate copies left 64 unique archived publications, and removal of 2 unrelated archiving errors left 62 publications for relevance assessment. Eighteen were excluded as outside scope, 17 of 44 eligible archived publications were not selected under the stated operational conditions, and 27 archived publications were retained. Seven additional included sources were not represented by a surviving archived PDF, yielding a final purposive narrative synthesis of 34 publications. Supplementary Table S2 provides the publication-level audit trail and archive exceptions. Contextual methodological and measurement references [22,23,24,25,26,33,34,35,36,37,38,39] are cited separately and are not counted in the 34-publication narrative synthesis.

**Figure 2:**
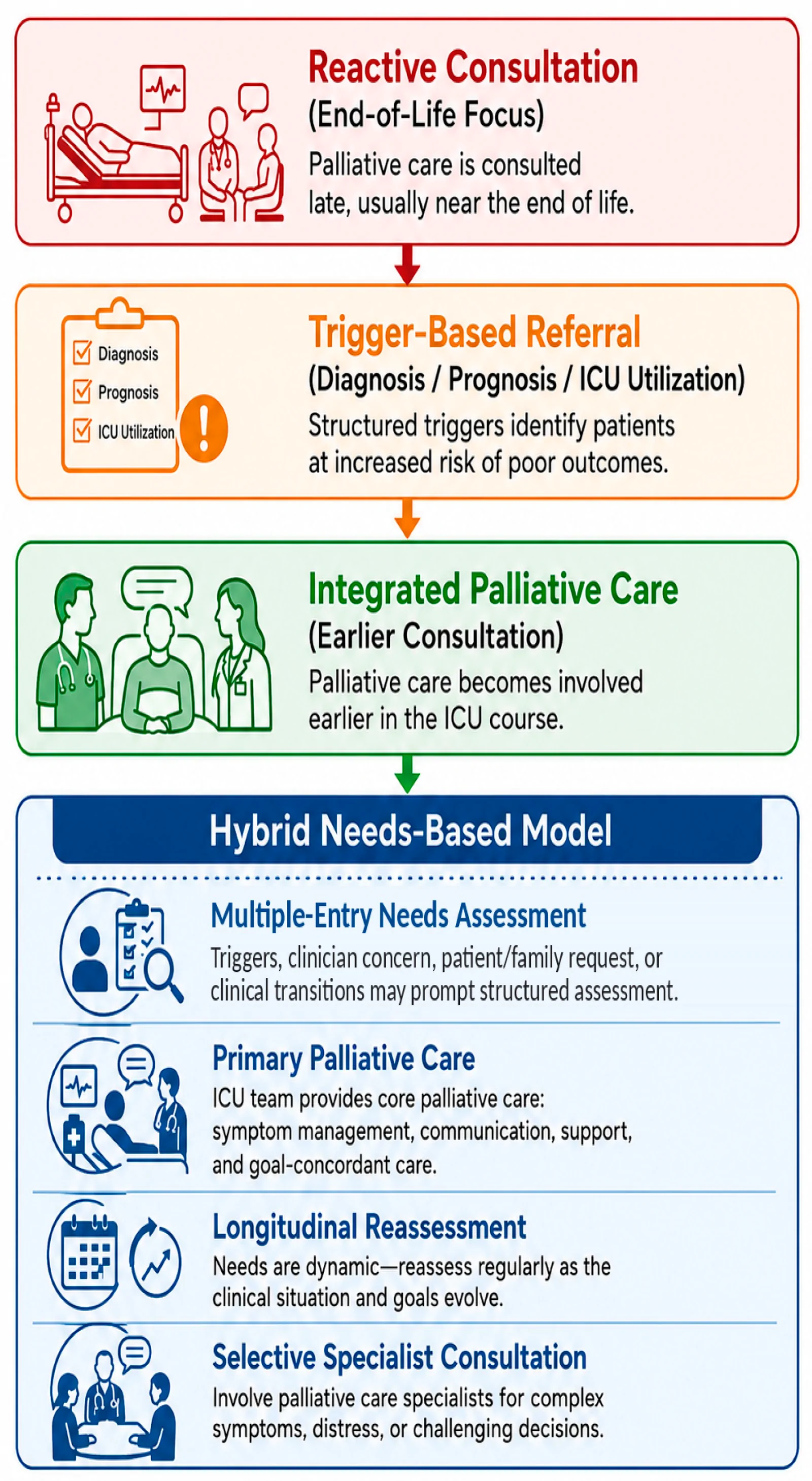
Evolution of palliative care integration in the intensive care unit. Trigger-based referral introduced reproducible identification of clinical vulnerability. Later work increased attention to direct assessment of patient and family needs and to ICU-delivered primary palliative care. The sequence depicts conceptual development in the literature, not evidence that one model is superior to another.

**Table 1:** Key milestones in the evolution of ICU palliative care integration.

Period	Key Development	Representative Sources	Contribution
2004–2010	Early ICU palliative care and end-of-life guidance	White 2004 [1]; Campbell 2006 [2]; Truog 2008 [3]; Nelson 2010 [4]	Defined core domains and service models
2007–2016	Communication and family-centered interventions	Lautrette 2007 [8]; Wright 2008 [9]; Carson 2016 [10]; Curtis 2016 [40]	Extended attention to family outcomes and communication
2013–2020	Trigger-based referral and implementation	Nelson 2013 [14]; Hua 2014 [15]; Hua 2018 [16]; Kistler 2020 [29]	Standardized identification of clinically vulnerable patients
2021–2022	Reassessment of trigger performance	Metaxa 2021 [18]; Cox 2022 [17]; Murali 2022 [44]; Curtis 2022 [19]	Raised questions about trigger status, measured need, and acceptability
2024–2025	Implementation and contextual adaptation	Salins 2024 [20]; Meddick-Dyson 2024 [21]; Ernst 2025 [45]	Emphasized implementation context and evidence gaps

**Table 2:** Trigger-based and needs-assessment approaches to ICU palliative care integration.

Dimension	Trigger-Based Approach	Needs-Assessment Approach
Primary purpose	Identify clinical vulnerability or predefined eligibility	Characterize reported or observed supportive care needs
Typical inputs	Diagnosis, prognosis, organ support, utilization	Symptoms, communication, psychological, family, decisional, social, and spiritual domains
Operational features	Simple, reproducible, and potentially automated	Directly focused on need but instrument- and respondent-dependent
Timing	Usually episodic	Potentially repeated; optimal frequency unknown
Resource implications	Lower screening burden; broad criteria may increase referrals	More staff time, training, and respondent participation
Key uncertainty	Validity as a proxy for multidimensional need	ICU-specific validity, proxy error, action thresholds, temporal responsiveness, and feasibility
Current evidence	Process and utilization evidence; limited direct validation against need	Instrument-specific measurement evidence; no validated referral threshold or comparative trial establishing superior outcomes

**Table 3:** Illustrative instruments relevant to multidimensional needs assessment in adult critical care and their principal limitations.

Instrument (Type)	Domains Covered	Respondent	Validation in Adult ICU	Principal Limitations in Critical Care
NEST (multidimensional needs screen)	Social, existential, symptom, and therapeutic domains	Patient or family	Applied in ICU; developed outside critical care [17,33]	Self-report and proxy constraints; no empirically derived referral threshold
IPOS/POS (outcome and needs measures)	Physical, psychological, practical, spiritual, and communication domains	Patient or staff proxy	We identified no formal validation for general adult ICU use [34]	Validation mainly in palliative care populations; recall and proxy limitations
ESAS (symptom intensity)	Physical and selected psychological symptoms	Patient	Not validated as a multidimensional ICU needs screen	Requires communication; limited domain coverage
CPOT/BPS (observational pain scales)	Pain only	Clinician observer	Validated for pain assessment in adult ICU populations [36]	Single domain; cannot assess communication, decisional, family, social, or spiritual need
FS-ICU-24 (family satisfaction)	Satisfaction with care and decision-making	Family	Developed and validated in ICU [37]	Measures satisfaction rather than need; often administered late or after discharge
CCFNI and derivatives (family needs)	Information, proximity, support, comfort, and assurance	Family	Developed for critical care families [38]	Does not assess patient-level need; length and respondent burden
QODD (quality of dying and death)	End-of-life experience	Bereaved family or clinician	Used in ICU and validated retrospectively after death [39]	Retrospective and decedent-restricted; cannot guide prospective identification

NEST, Needs Near the End-of-Life Care Screening Tool (termed the Needs at the End-of-Life Screening Tool in the Cox report); IPOS, Integrated Palliative care Outcome Scale; POS, Palliative care Outcome Scale; ESAS, Edmonton Symptom Assessment System; CPOT, Critical-Care Pain Observation Tool; BPS, Behavioural Pain Scale; FS-ICU, Family Satisfaction in the ICU; CCFNI, Critical Care Family Needs Inventory; QODD, Quality of Dying and Death. This table is illustrative of instrument categories rather than an exhaustive inventory.

**Table 4:** Principal ICU palliative care integration approaches and unresolved questions.

Approach	Entry Mechanism	Principal Strength	Central Uncertainty
Consultative	Clinician referral	Specialist expertise for complex symptoms, communication, or conflict	Variable or late referral and limited specialist capacity
Trigger-based	Predefined clinical criteria	Consistency, simplicity, and potential automation	Validity as a proxy for multidimensional need
Needs assessment	Direct assessment of patient or family need	Direct attention to multidimensional concerns	Instrument validity, respondent burden, feasibility, and effect on outcomes
Integrated proposition	One or more candidate entry points	Combines operational and patient-centered elements	No comparative validation; components, timing, thresholds, and staffing remain uncalibrated

The integrated approach is an author-generated research proposition assembled from established components; no comparative study has validated the proposed combination or sequence. ICU, intensive care unit.

## Data Availability

No new participant-level dataset was generated or analyzed. The reconstructed study-selection audit trail and the focused PubMed search export are available from the corresponding author on reasonable request.

## Supplementary Materials

The following supporting information can be downloaded at: https://www.mdpi.com/article/10.3390/healthcare14193199/s1, Supplementary Figure S1: Conceptual research architecture for comparative evaluation; Supplementary Table S1: Study characteristics and evidence matrix for the 34 publications in the purposive narrative synthesis; Supplementary Table S2: Publication-level audit trail for the reconstructed archive and final purposive synthesis.
