# Supplementary material for: Trigger-Based Referral and Needs Assessment in the Intensive Care Unit: A Narrative Review and Conceptual Synthesis
Source: Healthcare (Basel). 2026 Sep 28;14(19):3199. doi: 10.3390/healthcare14193199 (PMC13664902; doi:10.3390/healthcare14193199)
Supplement: Supplementary file 1 [file healthcare-14-03199-s001.zip › Supplementary_Figure_S1.pdf]

Supplementary Figure S1

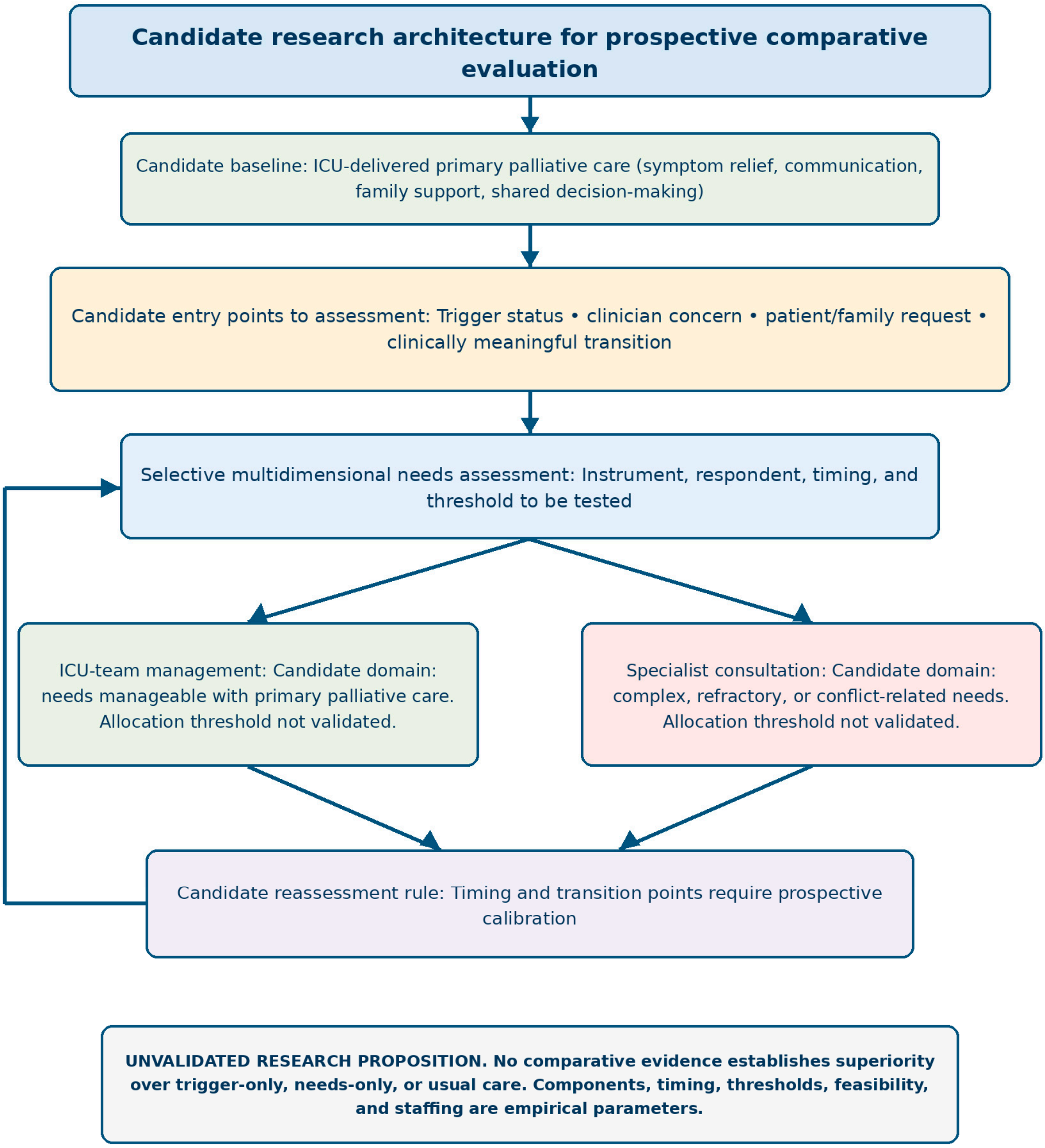

Supplementary Figure S1. Conceptual research architecture for comparative evaluation. The figure is an author-generated, hypothesis-generating research proposition rather than a clinical algorithm or validated model. It assembles candidate elements for prospective comparison: ICU-delivered primary palliative care, multiple possible entry points, selective multidimensional assessment, complexity-guided allocation, and reassessment. Instrument choice, respondent, timing, escalation thresholds, division of responsibility, feasibility, staffing, and resource effects remain unspecified empirical parameters. No comparative evidence currently establishes superiority over trigger-only, needs-only, consultative-only, fully integrative, or usual-care approaches.
