# Supplementary material for: Trigger-Based Referral and Needs Assessment in the Intensive Care Unit: A Narrative Review and Conceptual Synthesis
Source: Healthcare (Basel). 2026 Sep 28;14(19):3199. doi: 10.3390/healthcare14193199 (PMC13664902; doi:10.3390/healthcare14193199)
Supplement: Supplementary file 1 [file healthcare-14-03199-s001.zip › Supplementary_Table_S1.pdf]

Supplementary Table S1

Study characteristics and evidence matrix for the 34 publications in the purposive narrative synthesis

Ref.	Citation	Country/setting and design	Population or evidence base	Integration model or focus	Principal findings and contribution	Archive provenance
1	White & Luce (2004), Palliative care in the intensive care unit: barriers, advances, and unmet needs	United States; narrative review	Adult ICU literature	Barriers, unmet needs, consultative and integrative care	Describes symptom, communication, family, and organizational deficits; provides historical rationale for ICU palliative-care integration.	Archived: Article (5)
2	Campbell (2006), Palliative care consultation in the intensive care unit	United States; clinical review	Adult ICU practice	Specialist consultation	Summarizes indications and functions of specialist consultation and the need for earlier involvement.	Archived: Article (75)
3	Truog et al. (2008), Recommendations for end-of-life care in the intensive care unit	United States; multidisciplinary consensus statement	Adult ICU end-of-life care	Ethics, communication, symptom control, family support	Provides expert recommendations; used as guidance rather than independent evidence of effectiveness.	Archived: Article (70)
4	Nelson et al. (2010), Models for structuring a clinical initiative to enhance palliative care in the ICU	United States; expert project report	Adult ICUs and institutional programs	Consultative and integrative models	Defines alternative organizational models and implementation components for ICU palliative-care initiatives.	Archived: Article (59); duplicate copies removed
5	Aslakson et al. (2014), The changing role of palliative care in the ICU	United States; clinically focused review	Critically ill adults	Primary and specialist palliative care from ICU admission	Frames palliative care as need-based and concurrent with intensive treatment; summarizes implementation strategies.	Archived: Article (65); non-identical duplicate removed
6	Davidson et al. (2017), Guidelines for family-centered care in the neonatal, pediatric, and adult ICU	International; evidence-informed guideline	ICU patients and families; adult-relevant recommendations used	Family-centered care and shared decision-making	Supports structured communication, family presence, support, and participation; not treated as trial evidence.	Archived: Article (41)
7	Puntillo et al. (2010), Symptoms experienced by ICU patients at high risk of dying	United States; prospective observational study	171 communicative ICU patients at high risk of dying; 405 assessments	Direct symptom assessment	Documents frequent and distressing symptoms and illustrates both the value and respondent limitations of direct assessment.	No corresponding PDF in surviving archive; verified from indexed record and published abstract
8	Lautrette et al. (2007), A communication strategy and brochure for relatives of patients dying in the ICU	France; multicenter randomized trial	Relatives of patients dying in ICUs	Structured clinician-family communication	A structured conference and brochure improved selected family psychological outcomes; supports communication as a care intervention.	Archived: Article (43)
9	Wright et al. (2008), Associations between end-of-life discussions, care near death, and bereavement	United States; prospective cohort	Patients with advanced cancer and caregivers; non-ICU but transferable	Goals-of-care communication	Associates end-of-life discussions with less aggressive care and better caregiver bereavement adjustment; indirect ICU applicability.	Archived: Article (50)
10	Carson et al. (2016), Palliative care-led meetings for families of patients with chronic critical illness	United States; randomized clinical trial	Families of patients with chronic critical illness	Specialist-led family meetings	Did not demonstrate the expected improvement in family anxiety/depression and raised concern about unintended psychological effects; argues against assuming benefit.	Archived: Article (49)
11	Tran et al. (2016), Palliative care consultations in the Neuro-ICU	United States; qualitative study	Neuro-ICU clinicians and consultation practice	Consultation timing, roles, and barriers	Identifies uncertainty about roles, timing, and communication in a specialty ICU; informs implementation context.	Archived: Article (24)

Ref.	Citation	Country/setting and design	Population or evidence base	Integration model or focus	Principal findings and contribution	Archive provenance
12	Crooms & Gelfman (2020), Palliative care and end-of-life considerations for the frail patient	United States; clinical review	Frail critically ill adults	Frailty, goals, symptom burden, treatment decisions	Highlights needs and prognostic uncertainty in frailty; provides transferable clinical context rather than comparative evidence.	Archived: Article (26)
13	Berlin (2017), Goals of care and end of life in the ICU	United States; clinical review	Surgical and general critical care	Goals-of-care communication and end-of-life decisions	Summarizes practical communication and decision-making principles relevant to primary palliative care.	Archived: Article (10)
14	Nelson et al. (2013), Choosing and using screening criteria for palliative care consultation in the ICU	United States; expert advisory report	Adult ICU screening programs	Trigger selection and implementation	Provides candidate screening criteria and implementation guidance; establishes operational utility but not validity as a measure of multidimensional need.	Archived: Article (17); duplicate copy removed
15	Hua et al. (2014), Estimates of the need for palliative care consultation across US ICUs using triggers	United States; multicenter administrative analysis	Adult ICU admissions across US hospitals	Trigger-based estimation	Shows that the estimated consultation population varies with the trigger set and may represent a substantial service burden.	Archived: Article (55)
16	Hua et al. (2018), Derivation of data-driven triggers for palliative care consultation	United States; retrospective derivation study	Critically ill adults	Data-derived clinical triggers	Develops reproducible trigger criteria from clinical data; predicts eligibility/risk rather than directly measuring patient or family need.	Archived: Article (64)
17	Cox et al. (2022), Clinical palliative-care trigger status versus actual needs	United States; prospective multicenter cohort	Critically ill patients and family members	Conventional triggers compared with the NEST instrument	Found limited discrimination of instrument-measured unmet need by the evaluated triggers; single-instrument, single-trigger-set evidence requiring replication.	Archived: Article (63); non-identical duplicate removed
18	Metaxa et al. (2021), Palliative care interventions in ICU patients	International; systematic review	Adult ICU intervention studies	Consultative and integrative interventions	Finds heterogeneous interventions and outcomes with mixed patient, family, process, and utilization effects; does not establish one superior model.	Archived: Article (34); non-identical duplicate removed
19	Curtis et al. (2022), Integrating palliative care into the ICU	International; perspective	ICU palliative-care literature	Integrated care and future development	Provides conceptual synthesis and research priorities; treated as expert interpretation rather than comparative evidence.	Archived: Article (45)
20	Salins et al. (2024), Palliative-care practices in ICUs through appropriate-care concepts	International; umbrella review	Systematic reviews of ICU palliative-care practices	Appropriate care before, during, and after ICU admission	Maps broad practices and evidence gaps and emphasizes heterogeneity, context, and uncertainty.	Archived: Article (14)
21	Meddick-Dyson et al. (2024), Implementing palliative care in the ICU	International; systematic review with implementation mapping	Primary and specialist ICU palliative-care interventions	Implementation determinants and strategies	Identifies barriers, facilitators, mechanisms, and contextual dependencies; supports implementation analysis rather than model superiority.	Archived: Article (4)
24	Curtis et al. (2016), Communication facilitators to reduce family distress and treatment intensity	United States; randomized trial	Families of critically ill patients at high risk of death	Nurse-facilitated communication	Tests a structured communication intervention and informs the uncertain relationship between process improvement, family outcomes, and treatment intensity.	Archived: Article (30); duplicate copy removed
25	Quill & Abernethy (2013), Generalist plus specialist palliative care	United States; perspective	Serious-illness care across settings	Division of labor between generalists and specialists	Provides the conceptual basis for primary palliative care with selective specialist escalation; not a validated ICU allocation rule.	Archived: Article (61)

Ref.	Citation	Country/setting and design	Population or evidence base	Integration model or focus	Principal findings and contribution	Archive provenance
26	Oczkowski et al. (2016), Communication tools for end-of-life decision-making in the ICU	International; systematic review and meta-analysis	Adult ICU communication interventions	Decision aids and structured communication	Synthesizes effects on family and decision outcomes; heterogeneity limits conclusions about the optimal tool or delivery model.	No corresponding PDF in surviving archive; verified from indexed record and published abstract
27	Aslakson & Bridges (2013), Assessing ICU palliative care through patient-centered outcomes	United States; conceptual review	Adult ICU outcomes research	Patient-centered outcome selection	Argues for outcomes beyond mortality and utilization; informs evaluation domains rather than intervention effectiveness.	No corresponding PDF in surviving archive; verified from indexed record
28	Kistler et al. (2020), Triggered palliative-care consults	International; systematic review	Hospitalized and emergency-department populations, including critical care	Trigger-based consultation interventions	Shows heterogeneity in triggers, referral processes, and outcomes; supports operational feasibility but not universal trigger validity.	No corresponding PDF in surviving archive; verified from indexed record and published abstract
29	Khandelwal et al. (2015), Palliative-care interventions, advance care planning, and ICU utilization	International; systematic review	Studies of palliative care, advance care planning, and ICU use	Utilization effects	Summarizes heterogeneous evidence suggesting possible reductions in ICU use; secondary evidence was not treated as independent replication.	Archived: Article (22); duplicate copy removed
30	Ma et al. (2019), Early palliative-care consultation in the medical ICU	United States; cluster randomized crossover trial	High-risk medical ICU patients	Early triggered specialist consultation	Demonstrates feasibility and selected process/resource effects of early consultation; does not test the proposed integrated framework.	Archived: Article (2); duplicate copy removed
31	Murali et al. (2022), Clinicians' views on triggers for specialist palliative care in the ICU	United States; qualitative secondary analysis	ICU clinicians	Acceptability and implementation of triggers	Clinicians valued consistency but raised concerns about fit, over-referral, timing, and specialist capacity.	Archived: Article (73)
32	World Health Organization (2020), Palliative care	Global; authoritative organizational source	Definition and domains of palliative care	Multidimensional palliative-care concept	Provides definitional context only; not counted as empirical evidence of an ICU identification strategy.	Official web source; no archived PDF expected
33	Michalsen et al. (2019), Interprofessional shared decision-making in the ICU	International; systematic review and expert recommendations	Adult ICU shared decision-making	Interprofessional communication and decisions	Synthesizes evidence and recommendations for team-family decision-making; supports primary palliative-care competencies.	No corresponding PDF in surviving archive; verified from indexed record and published abstract
34	Ernst et al. (2025), Implementation of guidelines in palliative care	International; scoping review	Palliative-care guideline implementation across settings	Implementation determinants	Identifies organizational, clinician, and system factors affecting guideline uptake; ICU applicability is indirect.	Archived: Article (77)
35	Rao et al. (2022), Palliative and end-of-life care in ICUs in low- and middle-income countries	Low- and middle-income countries; scoping review	Adult ICUs in resource-constrained systems	Geographic transferability and implementation	Documents resource, workforce, cultural, and evidence gaps and cautions against assuming transferability from high-income systems.	No corresponding PDF in surviving archive; verified from indexed record and published abstract
36	Hua et al. (2020), Hospital-based palliative-care implementation and ICU use during terminal hospitalizations	United States; hospital-level observational study	Terminal hospitalizations across hospitals	Availability/implementation of palliative-care services	Associates implementation with lower ICU use in terminal hospitalizations; observational design does not identify the effect of a specific screening model.	Archived: Article (54)

Notes: This is a purposive evidence matrix, not a risk-of-bias table. Empirical studies, reviews, guidelines, consensus statements, perspectives, and organizational sources were used for different purposes as described in Section 2.5. References 22 and 23 informed narrative-review methodology and are not part of the 34-publication synthesis. Measurement-development references 37-43 are also reported separately in the manuscript. For sources without a surviving archived PDF, extraction was limited to information verifiable in indexed records, published abstracts, or the official organizational page.
