# Supplementary material for: Trigger-Based Referral and Needs Assessment in the Intensive Care Unit: A Narrative Review and Conceptual Synthesis
Source: Healthcare (Basel). 2026 Sep 28;14(19):3199. doi: 10.3390/healthcare14193199 (PMC13664902; doi:10.3390/healthcare14193199)
Supplement: Supplementary file 1 [file healthcare-14-03199-s001.zip › Supplementary_Table_S2.pdf]

Supplementary Table S2

Publication-level audit trail for the reconstructed archive and final purposive synthesis

Reconstructed counts: 73 archived PDF files; 9 duplicate copies removed; 64 unique publications represented; 2 unrelated archiving errors removed; 62 archived publications assessed; 18 scope exclusions; 44 eligible archived publications; 17 eligible non-selections; 27 archived publications retained; 7 additional included sources without a surviving archived PDF; 34 publications in the final synthesis.

A. Duplicate copies removed (n = 9)

Removed archive file	Retained archive file	Basis
Article (52)	Article (2)	Byte-identical duplicate
Article (74)	Article (17)	Byte-identical duplicate
Article (57)	Article (22)	Byte-identical duplicate
Article (44)	Article (30)	Byte-identical duplicate
Article (60)	Article (59)	Byte-identical duplicate
Article (60)[1]	Article (59)	Byte-identical duplicate
Article (23)	Article (65)	Same publication stored in a non-identical file
Article (47)	Article (34)	Same publication stored in a non-identical file
Article (69)	Article (63)	Same publication stored in a non-identical file

B. Unrelated archiving errors removed (n = 2)

Archive file	Bibliographic identity	Decision and reason
Article (68)	Hemofiltration in the early phase of sepsis: Friend or foe?	Unrelated to palliative-care identification or integration
Article (78)	Enteral versus parenteral nutrition in critical illness	Unrelated nutrition trial

C. Decisions for unique archived publications assessed (n = 62)

Archive file	Publication	Decision	Publication-specific reason	Retained representative source
Article (1)	Disparities in Palliative Care Among Critically Ill Patients With and Without COVID-19 at the End of Life	Excluded: outside scope	E2: Condition-specific COVID-19 cohort outside the comparative integration-model question	—
Article (2)	Early Palliative Care Consultation in the Medical ICU: A Cluster Randomized Crossover Trial	Included in synthesis	Retained as manuscript reference 30	—
Article (3)	Penguins and Palliative Care: Facilitating Cultural Change in the ICU	Eligible, not selected	C: Editorial with no distinct empirical contribution	Meddick-Dyson et al. [21]; Ernst et al. [34]
Article (4)	Implementing palliative care in the intensive care unit: a systematic review and implementation mapping	Included in synthesis	Retained as manuscript reference 21	—
Article (5)	Palliative care in the intensive care unit: barriers, advances, and unmet needs	Included in synthesis	Retained as manuscript reference 1	—
Article (6)	Palliative Care Clinicians' Views on Metrics for Successful Specialist Palliative Care Delivery in the ICU	Eligible, not selected	A: Narrow qualitative study of specialist-service metrics; implementation contribution covered more broadly	Meddick-Dyson et al. [21]
Article (7)	Current status of palliative care delivery and self-reported practice in ICUs in Japan	Excluded: outside scope	E4: Descriptive national status survey without comparative evaluation of an identification model	—
Article (9)	Pain Management Within the Palliative and End-of-Life Care Experience in the ICU	Excluded: outside scope	E5: Single-symptom clinical focus: pain management	—
Article (10)	Goals of Care and End of Life in the ICU	Included in synthesis	Retained as manuscript reference 13	—
Article (11)	Psychiatric and Palliative Care in the Intensive Care Unit	Eligible, not selected	A: General clinical review; relevant psychiatric and palliative domains covered by a broader retained ICU review	Aslakson et al. [5]
Article (12)	Impact of Palliative Care Screening and Consultation in the ICU: A Multihospital Quality Improvement Project	Eligible, not selected	B: Quality-improvement screening study represented in later intervention syntheses	Metaxa et al. [18]; Kistler et al. [28]
Article (13)	Compassionate Extubation of the ICU Patient and the Use of the Respiratory Distress Observation Scale	Excluded: outside scope	E5: Single procedural focus: compassionate extubation and respiratory-distress observation	—

Archive file	Publication	Decision	Publication-specific reason	Retained representative source
Article (14)	Assessing palliative care practices in intensive care units: an umbrella review	Included in synthesis	Retained as manuscript reference 20	—
Article (15)	Barriers and facilitators to palliative care implementation in the ICU: a qualitative systematic review	Eligible, not selected	A: Implementation barriers and facilitators covered by the retained systematic implementation review	Meddick-Dyson et al. [21]
Article (16)	Palliative care and end-of-life quality indicators in critical ICU patients: systematic review protocol	Excluded: outside scope	E3: Protocol without results	—
Article (17)	Choosing and Using Screening Criteria for Palliative Care Consultation in the ICU	Included in synthesis	Retained as manuscript reference 14	—
Article (18)	Integrating Palliative Care Into the ICU: From Core Competency to Consultative Expertise	Eligible, not selected	A: Conceptual levels of palliative-care expertise represented by retained organizational models	Nelson et al. [4]; Quill & Abernethy [25]
Article (19)	Hospice and Palliative Care for Patients in the Intensive Care Unit: Current Status in Countries Other than Korea	Excluded: outside scope	E4: Regional status report without comparative model evaluation	—
Article (20)	Palliative Care Principles and Anesthesiology Clinical Practice	Excluded: outside scope	E5: Specialty-focused review of anesthesiology practice; peripheral to the identification-model question	—
Article (21)	Palliative Care Needs in the Neuro-ICU	Excluded: outside scope	E5: Narrow Neuro-ICU needs review	—
Article (22)	Estimating the Effect of Palliative Care Interventions and Advance Care Planning on ICU Utilization	Included in synthesis	Retained as manuscript reference 29	—
Article (24)	Palliative Care Consultations in the Neuro-ICU: A Qualitative Study	Included in synthesis	Retained as manuscript reference 11	—
Article (25)	Palliative care in the ICU: relief of pain, dyspnea, and thirst	Excluded: outside scope	E5: Narrow symptom-management focus: pain, dyspnea, and thirst	—
Article (26)	Palliative Care and End-of-Life Considerations for the Frail Patient	Included in synthesis	Retained as manuscript reference 12	—
Article (27)	Utilization and Delivery of Specialty Palliative Care in the ICU	Excluded: outside scope	E4: Descriptive report of specialist-service utilization and delivery	—
Article (28)	Palliative Care in the Neurologic ICU—Are We There Yet?	Excluded: outside scope	E5: Narrow Neuro-ICU editorial	—
Article (29)	Clinical outcomes of a joint ICU and palliative care multidisciplinary rounding model	Eligible, not selected	A: Single-center joint-rounding cohort represented by broader intervention and implementation reviews	Metaxa et al. [18]; Meddick-Dyson et al. [21]
Article (30)	Randomized Trial of Communication Facilitators to Reduce Family Distress and Intensity of End-of-Life Care	Included in synthesis	Retained as manuscript reference 24	—
Article (31)	Retrospective Review of Trauma ICU Patients With and Without Palliative Care Intervention	Eligible, not selected	A: Narrow trauma-ICU cohort represented by the broader ICU intervention review	Metaxa et al. [18]
Article (32)	Guidelines for the Withdrawal of Life-Sustaining Measures	Excluded: outside scope	E5: Single procedural/ethical focus: withdrawal of life-sustaining measures	—
Article (33)	Focus on Palliative Care in the ICU	Eligible, not selected	C: Editorial summary with no distinct incremental evidence	Metaxa et al. [18]; Curtis et al. [19]
Article (34)	Palliative Care Interventions in Intensive Care Unit Patients	Included in synthesis	Retained as manuscript reference 18	—
Article (35)	Specialist Palliative Care Consultations in COVID-19 Patients in the ICU	Excluded: outside scope	E2: Condition-specific COVID-19 cohort outside the comparative integration-model question	—
Article (41)	Guidelines for Family-Centered Care in the Neonatal, Pediatric, and Adult ICU	Included in synthesis	Retained as manuscript reference 6	—
Article (42)	Who Is Attending? End-of-Life Decision Making in the Intensive Care Unit	Eligible, not selected	A: Narrow team-role study represented by the retained shared decision-making synthesis	Michalsen et al. [33]
Article (43)	A Communication Strategy and Brochure for Relatives of Patients Dying in the ICU	Included in synthesis	Retained as manuscript reference 8	—
Article (45)	Integrating Palliative Care into the ICU: A Lasting and Developing Legacy	Included in synthesis	Retained as manuscript reference 19	—
Article (46)	Early Palliative Care for Patients with Metastatic Non-Small-Cell Lung Cancer	Excluded: outside scope	E1: Non-ICU oncology population	—
Article (48)	Evidence-Based Palliative Care in the Intensive Care Unit: A Systematic Review of Interventions	Eligible, not selected	B: Earlier intervention systematic review superseded by later and broader syntheses	Metaxa et al. [18]; Salins et al. [20]
Article (49)	Effect of Palliative Care-Led Meetings for Families of Patients With Chronic Critical Illness	Included in synthesis	Retained as manuscript reference 10	—
Article (50)	Associations Between End-of-Life Discussions, Care Near Death, and Caregiver Bereavement	Included in synthesis	Retained as manuscript reference 9	—
Article (51)	Serious Illness Conversations in the Emergency Department for Older Adults	Excluded: outside scope	E1: Emergency-department population without an ICU integration focus	—

Archive file	Publication	Decision	Publication-specific reason	Retained representative source
Article (53)	Availability of Hospital-Based Palliative Care and Treatment Intensity for Critically Ill Patients	Eligible, not selected	B: Earlier observational hospital-availability analysis represented by the later implementation study	Hua et al. [36]
Article (54)	Implementation of Hospital-Based Palliative Care and ICU Use During Terminal Hospitalizations	Included in synthesis	Retained as manuscript reference 36	—
Article (55)	Estimates of the Need for Palliative Care Consultation Across US ICUs Using Triggers	Included in synthesis	Retained as manuscript reference 15	—
Article (56)	Potential Influence of Advance Care Planning and Palliative Care Consultation on ICU Costs	Eligible, not selected	A: Cost-focused modeling study without a distinct contribution to the identification-model question	Khandelwal et al. [29]; Salins et al. [20]
Article (58)	Integrated Palliative and Respiratory Care for Advanced Disease and Refractory Breathlessness	Excluded: outside scope	E1: Non-ICU advanced respiratory-disease service	—
Article (59)	Models for Structuring a Clinical Initiative to Enhance Palliative Care in the ICU	Included in synthesis	Retained as manuscript reference 4	—
Article (61)	Generalist plus Specialist Palliative Care—Creating a More Sustainable Model	Included in synthesis	Retained as manuscript reference 25	—
Article (62)	Palliative Care Versus Usual Care for Acute Critically Ill Emergency-Department Patients	Excluded: outside scope	E1: Emergency-department population without an ICU integration focus	—
Article (63)	Clinical Palliative-Care Trigger Status Versus Actual Needs	Included in synthesis	Retained as manuscript reference 17	—
Article (64)	Derivation of Data-Driven Triggers for Palliative Care Consultation	Included in synthesis	Retained as manuscript reference 16	—
Article (65)	The Changing Role of Palliative Care in the ICU	Included in synthesis	Retained as manuscript reference 5	—
Article (66)	High-Value Palliative Care in Critical Illness	Eligible, not selected	C: Letter commenting on the retained early-consultation trial	Ma et al. [30]
Article (67)	Developing Guidelines to Identify Surgical ICU Patients Who Would Benefit From Palliative Care	Eligible, not selected	A: Narrow surgical-ICU criteria study represented by broader trigger guidance and derivation work	Nelson et al. [14]; Hua et al. [16]
Article (70)	Recommendations for End-of-Life Care in the Intensive Care Unit	Included in synthesis	Retained as manuscript reference 3	—
Article (71)	Effects of a Palliative Care Trigger Assessment on Medical ICU Patient Outcomes	Eligible, not selected	A: Single-center doctoral quality-improvement project represented by retained trigger-intervention evidence	Kistler et al. [28]; Ma et al. [30]
Article (72)	Pediatric Palliative-Care Quality-Improvement Project in Neonatal Intensive Care	Excluded: outside scope	E1: Pediatric/neonatal population	—
Article (73)	Clinicians' Views on the Use of Triggers for Specialist Palliative Care in the ICU	Included in synthesis	Retained as manuscript reference 31	—
Article (75)	Palliative Care Consultation in the Intensive Care Unit	Included in synthesis	Retained as manuscript reference 2	—
Article (76)	Consensus Statements on End-of-Life Care in ICU: A Scandinavian Delphi Study	Eligible, not selected	A: End-of-life consensus contribution represented by broader retained consensus and shared-decision sources	Truog et al. [3]; Michalsen et al. [33]
Article (77)	Implementation of Guidelines in Palliative Care: A Scoping Review	Included in synthesis	Retained as manuscript reference 34	—

D. Included sources without a corresponding PDF in the surviving archive (n = 7)

Ref.	Included source	Archive exception and verification basis
7	Puntillo et al. (2010), Symptoms experienced by ICU patients at high risk of dying	No corresponding PDF in surviving archive; indexed record and published abstract used
26	Oczkowski et al. (2016), Communication tools for end-of-life decision-making in the ICU	No corresponding PDF in surviving archive; indexed record and published abstract used
27	Aslakson & Bridges (2013), Assessing ICU palliative care through patient-centered outcomes	No corresponding PDF in surviving archive; indexed record used
28	Kistler et al. (2020), Triggered palliative-care consults	No corresponding PDF in surviving archive; indexed record and published abstract used
32	World Health Organization (2020), Palliative care	Official web source; no archived PDF expected
33	Michalsen et al. (2019), Interprofessional shared decision-making in the ICU	No corresponding PDF in surviving archive; indexed record and published abstract used
35	Rao et al. (2022), Palliative and end-of-life care in ICUs in low- and middle-income countries	No corresponding PDF in surviving archive; indexed record and published abstract used

Operational codes: A = substantive contribution represented by a broader retained source; B = superseded or represented by a later review, larger study, or later analysis; C = commentary, letter, or conceptual item with no distinct incremental information for the review questions. Scope codes: E1 = non-adult-ICU setting or pediatric population; E2 = condition-specific cohort outside the comparative question; E3 = protocol without results; E4 = descriptive status or utilization report without comparative integration-model analysis; E5 = narrow clinical-domain, procedural, symptom, or specialty focus. Decisions were not based on the direction of findings.
